# All That Glitters Ain’t Gold: The Myths and Scientific Realities About the Gut Microbiota

**DOI:** 10.3390/nu17193121

**Published:** 2025-09-30

**Authors:** Priyankar Dey

**Affiliations:** Department of Biotechnology, Thapar Institute of Engineering and Technology, Patiala 147004, Punjab, India; priyankar.dey@thapar.edu or priyankardey28@gmail.com; Tel.: +91-9064275660

**Keywords:** microbiota, microbiome, dysbiosis, probiotic, fecal microbiota transplantation, diet

## Abstract

Gut microbial modulation through diet is central to human health and disease. Despite tremendous effort in understanding the impact of nutrients and drugs on the gut microbiota, and attempts to develop dietary strategies that facilitate gut-beneficial effects, several erroneous gut microbiota-associated concepts remain prevalent in popular belief. This article discusses widespread misconceptions about the gut microbiota, contrasting them with contemporary scientific facts. In this article, ten prevalent myths, including the obsolete 10:1 bacteria-to-human-cell ratio, the reductive categorization of microbes as ‘good’ or ‘bad’, and the discredited universal biomarker status of the Firmicutes/Bacteroidetes ratio in relation to metabolic diseases, have been debunked. Essential facts highlighting the context-dependency of the microbiome, considerable inter-individual heterogeneity, and dynamic reactivity to dietary changes are discussed. This questions the assumptions that increased diversity always signifies health, that probiotics are intrinsically safe, that fecal microbiota transplantation is a universal remedy, or that leaky gut syndrome constitutes a clearly defined diagnosis. It is highlighted that eubiosis and dysbiosis do not possess uniform criteria, and microbiome–drug interactions are extremely individualized. The gut microbiota operates as a dynamic, adaptive ecosystem, necessitating sophisticated, evidence-based methodologies for study and therapeutic application, transcending simplistic misconceptions in favor of tailored insights and therapies.

## 1. Introduction

**Figure 1:**
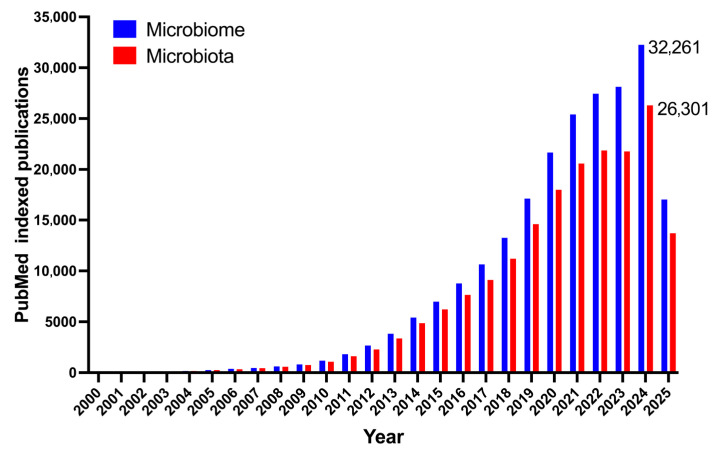
Yearly count of PubMed indexed articles containing the keyword ‘microbiome’ and ‘microbiota’.

The human gut microbiota, a complex ecology of trillions of microbes, significantly influences human health by affecting important processes including digestion, immunity, metabolism, and brain functions [1,2]. The unquestionable importance of microbiota has positioned it at the forefront of scientific research and attracted substantial public interest in recent years (Figure 1). This increase in attention has, regrettably, enabled the dissemination of both valid scientific discoveries and a plethora of oversimplified or completely inaccurate general notions. Numerous beliefs, previously regarded as universal truths or definitive solutions, have since been questioned or nullified by serious scientific inquiries. This article seeks to critically analyze and elucidate prevalent misconceptions regarding the gut microbiome, methodically differentiating them from proven scientific facts. This article aims to elucidate the complexity of the gut microbiota and its subtle consequences for health and disease by analyzing conventional narratives in light of contemporary research.

## 2. Core Concepts: Myths and Realities of the Gut Microbiome

### 2.1. Humans Harbor 100 Trillion Gut Bacteria

The often-used statistic of 100 trillion microbial cells in the human gut, typically coupled with the assertion that bacteria exceed human cells by a ratio of 10:1, has been fundamental to popular microbiome discussions. Recent and precise estimates, derived from extensive measurements, suggest that the total number of bacteria in the human body, primarily located in the gut, is approximately 38 trillion, which is roughly equal to the number of human cells, thereby disproving the longstanding 10:1 ratio [3]. The widely accepted assertion of around there being 10^14^ (100 trillion) bacteria in the human body came in the 1970s from a single ‘back-of-the-envelope’ estimate [3]. The original estimate assumed a uniform bacterial density across a 1 L gastrointestinal tract volume, which has now been shown to be erroneous, since bacterial density fluctuates considerably, with the colon serving as the principal site, with higher concentrations. Comprehensively updated estimations, using current data, determine the overall bacterial count in a 70 kg ‘reference human’ to be nearly 3.8 × 10^13^ (38 trillion) [3]. The new estimations for human cells indicate a count of an approximate 3.0 × 10^13^ (30 trillion). This new investigation revises the often-referenced 10:1 ratio, demonstrating that the bacterial count in the body is comparable to the quantity of human cells [3]. The aggregate mass of these microorganisms is estimated to be 0.2 kg. Although several sites continue to reference the range of 10–100 trillion or precisely 100 trillion [4], these estimates are derived from outdated or imprecise computations that have subsequently been substantially refined. This scenario demonstrates how an early, imprecise estimate, if widely circulated, may be established as a ‘fact’ despite the emergence of more exact evidence. The 10:1 ratio was notably awe-inspiring, which contributed to its wide and enduring adoption. The present comprehension, while still remarkable in scope, is more subtle and illustrates the ongoing refinement of scientific knowledge. This underscores the need to rigorously assess scientific assertions, even those that are broadly endorsed, and remain informed on the most recent research developments. It also highlights the difficulty of rectifying rooted popular beliefs, despite substantial scientific data.

### 2.2. Higher Gut Microbial Diversity Is a Universal Marker of a Healthy Gut

The prevalent notion that increased microbial diversity in the gut universally indicates wellness has become firmly established in general discourse. This viewpoint frequently results in the reductive assumption that an increased diversity of bacterial species inevitably correlates with enhanced health. Nonetheless, empirical evidence indicates that although frequently associated with favorable health outcomes in both pre-clinical and clinical cases [5,6], gut microbial diversity is not a definitive or universal indicator of a healthy gut [7]. The relevance is, in reality, highly context-dependent, and the specific functional capacities of microbial communities often outweigh the sheer enumeration of species [8]. Defining a ‘healthy microbiome’ requires a thorough examination of several elements associated with human health and the environmental influences affecting the microbiota [9,10]. It is commonly recognized that a singular, universal definition of a healthy microbiome is absent, and it is conceivable that many healthy microbial profiles may exist across individuals [11,12]. The gut microbiome significantly affects host physiology, with specific microbial species crucially altering host responses to dietary factors and influencing metabolic pathways. A decline in microbial diversity has been noted in individuals with obesity [13,14], suggesting an association; however, this does not confirm that diversity is the exclusive driver of health, nor does it imply a general causal relationship in which diversity alone governs overall well-being [15]. Moreover, studies suggest that diversity correlates with particular behavioral characteristics. For instance, persons possessing extensive social networks often display a more varied microbiome [16], while anxiety and stress correlate with diminished diversity [17]. This indicates that diversity frequently results from intricate interactions between the host and its environment, rather than being a sole measure of health. This viewpoint emphasizes that merely quantifying ‘diversity’ is frequently inadequate.

The characteristics of diversity, i.e., the presence of various species, their relative abundances, and their combined functional capabilities—along with their interactions within the physiological and environmental context of the host—are the factors that fundamentally characterize health. This insight contests the oversimplified ‘more is better’ paradigm regarding microbiome health, promoting a tailored and functional evaluation instead of dependence on a singular, universal parameter. It suggests that effective treatments should target specific functional outcomes or a contextual ecological equilibrium rather than simply striving to enhance sheer variety.

### 2.3. There Is a Universal Definition of Eubiosis and Dysbiosis

It is sometimes assumed that a singular, universally applicable definition exists for a ‘healthy’ (eubiotic) gut microbiome and its ‘unhealthy’ counterpart (dysbiotic), suggesting a distinct and uniform microbial signature for each condition. Eubiosis and dysbiosis are personalized and context-dependent concepts, lacking a general definition owing to the significant diversity in gut microbiota composition across the population [18,19]. The makeup of the gut microbiota demonstrates considerable intra-individual and inter-individual variability, which greatly complicates the formulation of a uniform criteria for a healthy microbiome [20]. This indicates that the definition of a balanced or ‘eubiotic’ condition might vary significantly across individuals. Dysbiosis is not a distinct disorder; it may be present in several forms, including deficient dysbiosis, putrefactive dysbiosis, fermentative dysbiosis, susceptibility dysbiosis, and fungal dysbiosis [21,22]. This underscores its complex character, beyond a mere concept of ‘imbalance.’ The functions of certain bacteria species are neither inherently ‘beneficial’ or ‘harmful’; their effects might differ based on the distinct gut environment and overall health condition of an individual [22]. A microorganism that is advantageous in one situation may be neutral or even harmful in another. The scientific community acknowledges the significance of a balanced gut microbiota for general health, and dysbiosis is increasingly associated with many diseases. Nevertheless, this acknowledgment does not imply a common compositional description. The rising trend in patents concerning dysbiosis underscores its increasing significance in disease research; however, a consistent classification of the condition remains absent [23]. This scenario underscores a critical barrier in microbiome research and personalized medicine, i.e., the difficulty of defining ‘healthy’ or ‘normal’ given the intrinsic variability across individuals. A functional definition, i.e., what the microbiome provides for the given host, is sometimes more relevant than a strictly compositional one representing which specific microorganisms are present. The term ‘core microbiome’ denotes microbial genomes consistently linked to certain genotypes or habitats, but it does not suggest a universally ‘healthy’ makeup [24]. This comprehension requires a transition from a uniform approach to gut health to individualized methods. Diagnostic and therapeutic strategies must include individual microbial profiles and their distinct interactions with the host, shifting away from standardized universal microbial compositions.

### 2.4. Gut Bacteria Are Simply ‘Good’ or ‘Bad’

A common oversimplification in public discourse classifies gut bacteria into binary categories of ‘good’ (useful) or ‘bad’ (pathogenic), suggesting that specific species are intrinsically one or the other. The scientific fact is that the function of certain bacteria in the gut microbiome is extremely context-dependent, influenced by intricate interactions within the distinct habitat of the host, food, and overall microbial community structure [25]. There is no universally ‘beneficial’ or ‘harmful’ bacterium [26]. Enhancing understanding of condition-specific microbial functions is actively challenging the notion of beneficial vs. harmful bacteria. A more thorough comprehension of their functions in many circumstances is necessary prior to their extensive therapeutic and clinical use [27]. *Akkermansia muciniphila* is a notable probiotic, often linked to advantageous metabolic benefits [28]. Nevertheless, it also presents an emerging cautionary tale, indicating that its effects may differ based on contextual factors [29,30,31]. It is now widely acknowledged that gut microbes cannot be universally categorized as beneficial or harmful species. Certain species may be advantageous or detrimental to one individual but not to another, and microbes may exhibit varying behaviors in different contexts [23,26]. The scientific consensus recognizes that a standard definition of a healthy microbiome is absent, and it is plausible that so-called healthy microbiome can have diverse phenotype [32]. This further diminishes the concept of permanent ‘good’ or ‘bad’ species, since their impact on health is contingent upon the total ecosystem. An oversimplified classification may overlook the intrinsic diversity of the human microbiome and perhaps foster new stereotypes and stigma [11]. This notion suggests that the microbiome operates as a complex ecosystem, whereby the overall impact of a specific species is influenced by its complicated interactions with other bacteria, the host’s genetics, nutrition, and the surrounding environment. A species may favorably impact a balanced environment, although it may become detrimental if its population is uncontrolled or if the ecosystem is somehow disrupted. Indeed, recent studies indicate that colonization success, pathoadaptive mutation, and evasion of immune response can lead to opportunistic infections, even by the gut commensals [33]. This perspective redirects attention from attributing blame or commendation to specific species towards grasping the complex network of microbial interactions. This is essential for formulating effective microbiome therapies as well. Rather than focusing on eliminating pathogenic bacteria or only introducing helpful bacteria in isolation, efforts should emphasize the restoration of microbial ecological balance, the enhancement of advantageous functions, and the comprehension of the unique context of dysbiosis. This also informs public education, promoting a more sophisticated understanding of the gut microbial functions.

### 2.5. The Firmicutes to Bacteroidetes Ratio Universally Predicts Metabolic Disease

For a long time, the ratio of the two principal bacterial phyla in the gut, Firmicutes (F) and Bacteroidetes (B), was extensively advocated as a universal marker, especially concerning obesity and metabolic disorders. A higher F-to-B ratio (F/B) was often identified as having a conclusive association with obesity [34]. The F/B is an unreliable and arbitrary tool for evaluating gut health or forecasting metabolic illness, lacking general applicability [35]. A thorough meta-analysis of ten independent pieces of research examining the gut microbiota in obese individuals determined that the frequently employed F/B ratio is an arbitrary metric [35]. This finding directly undermines its efficacy as a dependable prediction or diagnostic instrument. The F/B ratio is deemed a unreliable measure of the functional characteristics of the gut microbiota and their effects on the host [35]. Moreover, its interpretation may be deceptive, as the relative abundance of specific advantageous members of the Firmicutes phylum, such as Lactobacillus, has been demonstrated to diminish with high-fat diets [36], thereby complicating any direct interpretation of this ratio concerning health or disease [35]. Research examining alterations in the F/B in anorexia nervosa has yielded contradictory outcomes, frequently hindered by methodological discrepancies, limited sample sizes, and inadequate data, hence constraining the generalizability of findings derived from this ratio [8]. Although these two phyla collectively represent around 80–90% of the gut microbiota, and their ratio is regarded as an indicator of bacterial diversity and health, the actual range seen in healthy individuals from Western European populations is quite wide, ranging from 1.3 to 10.3 [8]. This enormous variety, further impacted by the age of the individuals [37], highlights its constraints as an accurate universal indicator. This scenario illustrates a prevalent flaw in scientific investigation, i.e., the simplification of a very intricate biological system, such as the gut microbiome, comprising thousands of species and millions of genes, to a singular, readily quantifiable ratio. Although this simplification may seem attractive, it frequently neglects to reflect the genuine functional dynamics and the considerable inter-individual variability present in the system. The F/B ratio may indicate general trends, although it fails to represent the precise metabolic functions or host interactions that truly characterize health or disease conditions [38]. This acts as a warning against the oversimplification of intricate biological processes into easily quantifiable yet functionally insignificant measurements. Subsequent research and therapeutic applications must advance beyond simplistic ratios to more sophisticated, functional, and individualized evaluations of the microbiome.

### 2.6. Probiotics Are Always Safe and Universally Beneficial

Probiotics are often seen as generally safe dietary supplements that consistently provide health advantages, frequently without sufficient attention to particular strains, doses, or individual health conditions. Probiotics, while often advantageous, are not universally safe and may induce undesirable consequences such as bacteremia; in some circumstances, they may also aggravate or be associated with small intestine bacterial overgrowth (SIBO) [39]. Their effectiveness is greatly dependent on strain and circumstance. A review of probiotic intervention studies indicated that although randomized controlled trials (RCTs) typically demonstrated no statistically significant elevation in the risk of overall adverse events, several case studies reported fungemia and some bacteremia that were potentially linked to administered probiotic organisms [40]. This risk is especially seen in those with impaired health [41].

The association between probiotics and SIBO is intricate and multifaceted. Clinical studies have shown that probiotics may enhance the effectiveness of SIBO treatment when used with antibiotics, particularly in at-risk populations such as children and pregnant women [42]. Twenty-four specific strains, such as *Saccharomyces boulardii,* have shown efficacy in the elimination of SIBO and alleviation of symptoms [42]. Contradictory research indicates that certain probiotics may aggravate SIBO symptoms or hinder diagnosis [39]. A study revealed that supplementation with *Bifidobacterium infantis* 35624 markedly elevated methane excretion to levels often regarded as indicative of SIBO diagnosis, hence implying a potential distortion of breath test precision [43]. Additionally, a condition marked by cognitive fog, flatulence, and abdominal distension has been associated with probiotic use, SIBO, and D-lactic acidosis, with symptom alleviation seen after the cessation of probiotics and the administration of antibiotics [44]. Individuals suffering from brain fog had a much higher propensity for probiotic use [44]. At present, no singular probiotic intervention possesses enough evidence to warrant universal recommendation for all patients with SIBO [43]. This highlights the essential need for future research to ascertain which strains, as well as under what circumstances and at what dosages, may be really advantageous. This intricate array of studies indicates that probiotics are not universally homogeneous. Their effects are extremely unique to the strain and are significantly influenced by the health of the host and existing gastrointestinal conditions [41,45]. Although they can function as effective therapeutic strategies in particular scenarios, such as recurrent *Clostridioides difficile* infections or as adjuncts in the treatment of SIBO, long-term use also pose inherent risks to susceptible populations and may exacerbate certain conditions or disrupt diagnostic processes if not judiciously chosen [46]. The concept of probiotics as ‘safe and universally advantageous’ is therefore challenged by this complex reality. This requires a more careful and individualized method of probiotic delivery. Healthcare practitioners must evaluate the individual patient’s health, possible susceptibilities, and the exact strain and dose of the probiotic, avoiding generic suggestions. It underscores the need for meticulous and systematically documented safety evaluations in any probiotic intervention research. Moreover, compliance with ISAPP recommendations is crucial since they offer a consistent, evidence-based framework for the application of probiotics [47]. This would guarantee strain-specific effectiveness, suitable doses, and safety evaluations, therefore reducing dangers such as bacteremia or SIBO that may occur due to unregulated or improper probiotic use in vulnerable persons.

### 2.7. Gut Microbial Effects on Drugs and Nutrients Are Uniform Across Individuals

It is often thought that drugs and nutrients would have uniform effects in all individuals, neglecting the considerable inter-individual heterogeneity shaped by individual microbial makeup. The scientific fact is that the gut microbiota significantly affects individual reactions to medications and foods, modifying their bioavailability, bioactivity, and metabolism, resulting in highly individualized results. The relationship between gut microbiota and frequently used non-antibiotic medications is intricate and reciprocal [48]. Pharmaceuticals may modify gut microbial populations, whereas the varied gut microbiota can influence medication’s effectiveness by enzymatically modifying the structure of the drug, thereby affecting its bioavailability, bioactivity, or toxicity, a discipline referred to as pharmacomicrobiomics [49]. This microbial effect may indirectly affect an individual’s reaction to treatments like cancer immunotherapy [50]. Individual responses to dietary intake exhibit significant variability, mostly due to variances in the microbiota and host characteristics [15]. The makeup and function of the gut microbiota directly affect the metabolic response to food, making customized nutrition an increasingly prominent area of study [51]. Comprehending drug metabolism through the microbiota and its impact on nutritional utilization is essential for discovering new methods to modulate gut bacteria, hence enhancing therapeutic efficacy and clinical results in precision medicine [48]. The acknowledged adaptability of the gut flora indicates the possibility of therapeutic modifications to enhance individual reactions to certain food elements [51]. These studies jointly establish the gut microbiota as a vital, dynamic modulator that influences an individual’s processing of external inputs, including pharmaceutical medicines and nutritional components. This indicates that a ‘standard dose’ of medicine or a broad ‘healthy diet’ might significantly vary results across individuals, positioning the microbiota as a crucial factor in attaining customized healthcare. The ability to regulate the microbiome enhances its promise in precision medicine. This knowledge requires a paradigm change in pharmacology and nutrition, transitioning from population-level averages to individualized therapies. Integrating microbiome data into clinical practice may allow for medicine doses enhanced, adverse responses forecasted, and food advice customized to achieve better health results.

### 2.8. Leaky Gut Syndrome Is a Clearly Defined Clinical Diagnosis

The phrase leaky gut syndrome is often used in popular media and by alternative health practitioners to denote a distinct medical condition marked by heightened intestinal permeability, typically attributed as the underlying cause of other apparently unrelated symptoms and ailments.

In scientific terms, although increased intestinal permeability (or intestinal barrier dysfunction) is acknowledged as a physiological occurrence associated with the development of numerous intestinal and extra-intestinal diseases [52,53], the concept of leaky gut syndrome as an independent, universally recognized clinical diagnosis with a definitive etiology and standardized diagnostic criteria is frequently oversimplified and lacks substantial scientific agreement [54]. The integrity of the gut barrier is essential for preventing hazardous and pyrogenic metabolites from entering the circulation [55]. Factors influencing this include stress, an unhealthy diet, excessive alcohol intake, and antibiotic use may actually undermine this barrier, resulting in enhanced intestinal permeability [55]. This is scientifically linked to several chronic diseases, including inflammatory bowel disease (IBD), irritable bowel syndrome (IBS), obesity, type 1 diabetes mellitus, and celiac disease. Particular microbial alterations, including a decrease in beneficial short-chain fatty acids (SCFA) and an elevation in proinflammatory microorganisms, might provoke harmful modifications that undermine intestinal barrier integrity [54]. The restoration of a healthy gut microbiota and the enhancement of intestinal barrier function constitute a critical focus of current microbiome research. Interventions such as ketogenic and Mediterranean diets, together with probiotics, prebiotics, synbiotics, and postbiotics, have shown effectiveness in reinstating advantageous microbial communities, improving tight junction protein expression and functionality, and diminishing intestinal permeability [56,57]. This distinction is essential for clinical practice and patient education. The scientific literature recognizes the phenomena of heightened intestinal permeability and its evident significance in disease etiology. Nonetheless, it does not recognize leaky gut syndrome as a separate, main diagnosis in the same way it categorizes Crohn’s disease. The prevalent syndrome suggests a unique, overarching etiology for several symptoms, which oversimplifies a complicated physiological process that may be a result of or contributor to multiple diseases, rather than an independent disease. Concentrating on enhanced intestinal permeability facilitates evidence-based therapies that address underlying causes or contributing variables, such as dysbiosis. Conversely, naively accepting leaky gut syndrome may result in unsubstantiated and possibly useless or detrimental diagnostic or treatment methods. This highlights the need for accurate scientific language in medical communication.

### 2.9. Fecal Microbiota Transplantation (FMT) Is a Universal Cure-All

In light of its notable efficacy in addressing certain diseases, FMT is often exaggerated as a miracle cure for a broad range of ailments. This has regrettably resulted in inflated expectations and the widespread emergence of uncontrolled DIY methods [58]. FMT is a useful and transforming treatment for some diseases, including recurrent *Clostridioides difficile* infection (CDI) [59]; however, it is not an absolute solution and has inherent dangers, limits, and ethical problems [60,61]. FMT has shown high effectiveness for recurrent or refractory CDI, with notable cure rates (e.g., 90% in a real-world registry research) and a mostly good short-term safety profile [62]. It is highly recommended as the preferred therapy for recurring CDI [63]. Although its use is broadening to include more gastrointestinal and extra-intestinal disorders, research in these domains is still under progress, and substantial data endorsing its extensive usage remains scarce for several ailments [11]. Awareness of the adverse occurrences linked to FMT has increased as its use has expanded [64]. These may include gastrointestinal symptoms such as diarrhea and abdominal discomfort, and, less often, more serious consequences such as hospitalization. A known risk of disease transmission exists if donor-screening measures are insufficient. A small number of patients have reported new diagnoses of diseases such as irritable bowel syndrome or inflammatory bowel disease after FMT, underscoring the need for long-term safety follow-up studies [62]. The idea of FMT as a panacea may result in its abuse, as shown by the promotion of unregulated DIY home-FMT kits [11]. This commercialization and associated excitement, sometimes driven by social media, might generate excessive expectations among the public and may ultimately undermine credible microbial research [11]. This scenario exemplifies the intrinsic conflict between revolutionary scientific advancements and societal perception. Although FMT is a potent therapeutic instrument, its use must be carefully directed by robust data and a comprehensive grasp of its particular risks and benefits for specified applications. The cure-all fallacy stems from an overgeneralization of success in one domain to other domains, neglecting the intricacies of illnesses’ pathophysiology and the possibility of unforeseen repercussions within a highly customized microbial environment. This underscores the essential function of evidence-based medicine in overseeing innovative medicines and accentuates the need for the responsible dissemination of scientific discoveries to the public to regulate expectations and avert the abuse of potentially beneficial treatments.

### 2.10. The Gut Microbiome Is Static and Difficult to Change

Certain observations indicate that the gut microbiota is mostly established during early infancy and is resistant to substantial or rapid modification, suggesting that food or lifestyle changes may have relatively little influence. The scientific truth is that the gut microbiota is very dynamic and may swiftly and consistently react to environmental variables, especially dietary alterations, showcasing its extraordinary adaptability [65]. The quick ingestion of meals exclusively consisting of animal or plant sources may swiftly modify microbial community composition and may even surpass individual variations in microbial gene expression [65]. These substantial alterations may manifest as soon as one day after a dietary modification. Diet-induced alterations signify essential functional transformations within the microbial community, paralleling distinctions seen between herbivorous and carnivorous animals, and including trade-offs between glucose and protein fermentation [65,66]. An animal-based diet has been shown to enhance the prevalence of bile-tolerant microbes while diminishing those that metabolize dietary plant polysaccharides [67]. The rapid flexibility of the gut microbiota likely enhances the variety of human eating practices. This intrinsic dynamic is essential for comprehending how nutrition significantly impacts both health and illness conditions. The acknowledgment that gut microbiota could be influenced implies that it may be medically modified to affect individual responses to dietary strategies, thereby establishing a fundamental basis for precision nutrition initiatives [51]. This comprehension immediately debunks the static perspective, portraying the microbiome as a highly flexible and adaptive ecosystem. The rapid adaptability of the gut microbiota to dietary changes allows nutritional strategies to have quick and substantial effects, establishing it as a potent and accessible target for health regulation. Diet may significantly impact microbial composition, perhaps surpassing inherent inter-individual variances. This comprehension empowers both people and physicians, signifying that dietary and lifestyle alterations are not ineffective but rather powerful instruments for influencing gut health. This underscores the significance of nutrition as a principal determinant of microbiome makeup and function, while also presenting substantial opportunities for dynamic, real-time treatments to enhance health outcomes.

## 3. Conclusions

**Figure 2:**
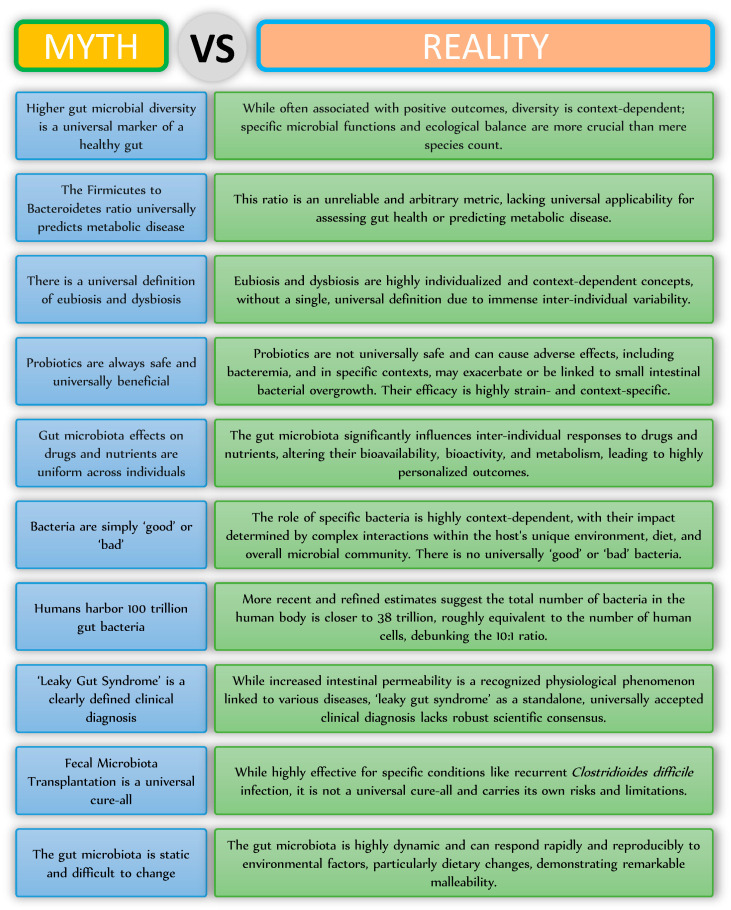
Myths and realities related to the gut microbiota.

The human gut microbiota is a complex, dynamic, and highly personalized ecology. The undeniable and extensive influence on host health is fundamental to contemporary biological knowledge, although our understanding of it is continually advancing, progressing beyond the basic narratives that have often infiltrated public discourse. This narrative piece has methodically elucidated various common misconceptions regarding the gut microbiota, illustrating that notions such as universal health markers, static microbial ratios, or simplistic good/bad classifications are frequently significant oversimplifications that do not accurately reflect the intricate nature of microbial interactions (Figure 2). This scientific reality is defined by context-dependency, considerable inter-individual variability, and the notable plasticity of the microbial population. As research advances, a more profound understanding of particular functional pathways, complex host–microbe interactions, and individualized responses will be essential. This enhanced understanding will facilitate the creation of more accurate diagnostic instruments and focused therapeutic strategies, advancing the scientific and medical fields toward fully using the gut microbiome to enhance human health and prevent chronic diseases.

## Data Availability

Not applicable for perspective.
